# Effective Teacher Professional Development for School-Based Mental Health Promotion: A Review of the Literature

**DOI:** 10.3390/bs14090780

**Published:** 2024-09-05

**Authors:** Hannah L. Dinnen, Nicole S. Litvitskiy, Paul D. Flaspohler

**Affiliations:** 1 Department of Behavioral Medicine and Clinical Psychology, Cincinnati Children’s Hospital Medical Center, Cincinnati, OH 45225, USA; 2 Department of Psychology, Miami University, Oxford, OH 45056, USA; litvitns@miamioh.edu (N.S.L.); flaspopd@miamioh.edu (P.D.F.)

**Keywords:** school mental health, professional development, technical assistance, training, prevention

## Abstract

Teachers are critically involved in the delivery of school-based mental health promotion (SMHP) interventions in school, though pre-service teacher education often leaves teachers feeling underprepared in this area. Thus, understanding how best to build teachers’ capacity for delivery through effective professional development (PD) is essential for teachers to fulfill their role as delivery agents to achieve SMHP outcomes. This systematized review focuses on identifying components of high-quality teacher PD for SMHP and examining the empirical support for these components. In a two-phase analysis, we examined the descriptive literature regarding teacher PD for SMHP to identify common elements. This resulted in the identification of components relevant to training (interactive, guided by staff input) and technical assistance (TA), including the goals (skill development, motivation, generalization/adaptation), approach (collaborative, individualized, data-driven, strengths-based), and activities (modeling, performance feedback, reflection, problem solving) involved. A second phase focused on the examination of empirical evidence for these PD components. While the empirical evidence for these components was limited, the identified components represent a current standard of practice in teacher PD for SMHP, based on the existing research. These components may provide a framework for practical use in planning teacher PD related to SMHP and for designing future research into effective capacity building in this area.

## 1. Introduction

Mental health concerns impact up to a quarter of children and adolescents in the United States [1,2], and schools provide a logical and accessible point for supporting youth mental health [3]. School mental health promotion (SMHP) encompasses a full continuum of preventative and responsive mental health, including universally delivered preventative interventions [3,4]. These universal prevention programs and practices, which focus on the positive development of all students, can be effective [5,6,7,8], but strong implementation is needed for results [7,8,9]. Successful implementation requires effective professional development for teaching staff responsible for implementation [10,11,12]. However, it is not clear what effective professional development for teachers in this area entails [13,14,15]. The following paper presents a systematized review of the literature regarding effective teacher professional development for school mental health innovations, and how this may inform current practices and future directions. 

### 1.1. Teachers’ Role in SMHP

With teachers spending approximately 1080 h a year with a group of children, teachers have significant opportunities to support SMHP and enhance students’ mental health through positive interactions, the school environment, and the curriculum [16]. Unsurprisingly, teachers are centrally involved in SMHP efforts, and in particular in the delivery of universal prevention innovations. While programs are sometimes delivered by psychologists or other specialists such as school counselors, teachers are often involved in the delivery of these services [15,17]. Teachers are actively involved in up to 40.8% of interventions, and are particularly likely to be involved in delivering universal (Tier 1) school mental health interventions [18]. Teachers are especially well positioned to deliver universal interventions targeting all students because they are able to connect mental health innovations into general curricula, provide additional opportunities for students to practice and generalize skills, and integrate mental health promotion into the broader classroom and school environment [19]. Thus, teachers are often key in the delivery of universal prevention interventions. 

While teachers are often involved in delivering universal prevention interventions, evidence on the effectiveness of their involvement relative to other potential providers such as school mental health professionals is mixed. Some reviews have found similar program outcomes regardless of the personnel implementing the intervention [8,18], while others have found teachers to be less effective than mental health specialist staff [8,20]. Some research has supported stronger implementation quality from teachers compared to outside agency specialists, although this finding has not been consistent [21,22]. 

While it seems that teachers’ success in implementing these innovations is mixed, there is limited research on the individual teacher and contextual factors that influence implementation and sustainability [19]. Teacher receptivity has been suggested to be an important influence on successful innovation delivery [9,19]. Teachers’ own mental health, including stress, burnout, and work-related efficacy, may also impact the success of their delivery [19,23,24]. Finally, researchers have identified training and the receipt of ongoing support as important influences on successful innovation delivery [7,9]. Teachers need to understand the theory, purpose, and core components of an innovation to effectively deliver and appropriately adapt it [9]. Taken together, the existing evidence indicates that teachers may be effective agents in delivering innovations with the appropriate preparation and support. 

### 1.2. Teacher Preparation for SMHP

While a variety of factors may influence teachers’ effectiveness as SMHP delivery agents, one prominent barrier is teachers’ limited preparedness in this area. Research on standards and curriculum for pre-service teacher education indicates that pre-service teacher candidates may receive insufficient preparation in the area of mental health. In a review of pre-service teacher education, results suggested that pre-service education for teachers lacked specific, competency-based training regarding mental health principles and practices and that coursework in this area was generic and not focused on practical application [25]. In a review of teacher certification standards, while all state certification standards across U.S. states included at least some reference to mental health, the standards related to student mental health varied substantially across states and tended to be vague, with little specific guidance regarding the mental health skills or knowledge teacher candidates should acquire [26]. In particular, fewer than half of states have standards addressing preventative intervention [26]. The broad inclusion of SMHP-related teacher competency standards across states reflects that mental health is an important priority, but the pre-service teacher standards do not lay out the specifics needed for teacher education programs to consistently prepare teachers in this area [27]. This is reflected in the pre-service teacher curriculum. In a review of curriculum requirements for pre-service elementary teachers in a set of randomly selected college/university teacher education programs, researchers found limited content, objectives, or assignments related to social, emotional, or behavioral problems [28]. Fifteen percent of the sample did not address these topics at all in required coursework. On average, the researchers calculated actual time spent on class topics, based on course syllabi, included only three hours total of pre-service education related to intervention, and an average of just one hour spent on classroom management. These results indicate that, in practice, pre-service teacher candidates often receive little preparation in the area of social, emotional, and behavioral problems, which impacts their preparedness to implement innovations designed to promote school mental health. 

Teachers in the field also report a lack of training in the area of SMHP and a desire for additional information and training in this area. Several studies of teachers’ perspectives support that while many teachers view addressing mental health needs as a necessary part of their role, they do not feel adequately prepared to do so [29,30,31,32]. In one study, teachers reported that lack of information and training was the greatest barrier for them in addressing students’ mental health problems [32]. These teachers also demonstrated limited knowledge of and self-efficacy regarding mental health issues on brief survey assessments. Teachers both recognize the need for more information and training and report a desire for PD in this area [30,32]. If teachers do not feel adequately prepared to address these issues, they may feel burdened by students’ mental health needs, which in turn has been associated with students in their classroom reporting more negative feelings (i.e., frustration and anger) about school, lower feelings of personal esteem, and lower feelings of academic efficacy [31]. Although most teachers express a desire to support student mental health needs, insufficient pre-service training regarding SMHP may negatively impact their ability and confidence to do so.

It is also important to acknowledge that teachers are asked to take on many roles and responsibilities, and not all teachers are eager to take on a role in SMHP. Qualitative studies reveal that support for teacher involvement in addressing mental health needs is not universal, with some teachers reporting that they themselves or their colleagues may not view addressing these issues as part of a teacher’s job, that they are not comfortable or qualified to address these concerns, that teachers’ own unaddressed emotional needs may make it difficult for them to address these issues, and that there may be confusion regarding the delineation of roles and professional boundaries regarding the provision of school mental health services [33,34]. Thus, addressing the perspectives and support needs of these teachers is likely an important consideration in order for teachers to participate in SMHP. 

### 1.3. Capacity Building Is Critical for Implementation

To actualize the benefits of SMHP, implementation is critical [7,8,9]. While many schools and other organizations operate from a research-to-practice approach in which they seek evidence-based interventions that have yielded positive results under research conditions [10,11,35], without attention to implementation, even programs with research support often are not successful in new local settings [10,12]. This is particularly true of “operator-dependent” innovations which rely on practitioners for delivery, such as SMHP innovations [36] (p. 364). In particular, building practitioners’ capacity to deliver an innovation has been identified as an important component in bridging the gap between research and practice [10,11,35,37,38]. Building both general capacity (i.e., infrastructure, general skills, motivation) and innovation-specific capacity (i.e., skills and knowledge to carry out the specific innovation) at all levels contributes to the successful and enduring implementation of prevention innovations [11,35,39]. With teachers serving in an important role in SMHP but often receiving limited pre-service training, effective SMHP implementation requires developing teachers’ capacity to successfully deliver these innovations.

Establishing a support system that includes pre-intervention, research-based training, and ongoing support for all those in the school community involved in implementation is often cited as critical to SMHP innovation outcomes [21,40]. The provision of training and technical assistance (TA, i.e., ongoing support, consultation) are consistently identified as crucial for successful implementation sustainability [22,41]. Notably, there are a variety of terms for the training and TA activities used to develop teachers’ practical application capacity, including workshops, coaching, mentoring, and others. To capture this range of activities, we refer to all training and TA activities aimed at developing capacity for implementing an innovation within the umbrella term professional development (PD). PD refers more generally to all types of facilitated learning activities for current teachers to enhance their professional capacity [42]. The general goal of teacher PD in the area of SMHP is to prepare teachers and other school providers to effectively complete the tasks required for school mental health related innovations, including developing the relevant innovation-specific skills but also addressing expectations, motivation, and self-efficacy [19,21,43]. However, while the general goals of PD in this area are clear, there is limited research regarding the specific characteristics of effective PD for teachers in the area of SMHP [13,14,15].

### 1.4. Effective Teacher PD for SMHP

A growing body of research regarding teacher PD related to academic instruction exists and suggests patterns among effective PD programs. Teacher PD research has largely focused on preparation for math, science, and literacy instruction, emphasizing students’ academic outcomes [42,44,45]. In PD targeting these areas of academic instruction, researchers have consistently identified a number of characteristics of effective PD [44,46,47,48], including a focus on specific content; the use of active learning strategies; adequate duration and follow-up; the support of collaboration; providing coaching; and the use of high-quality, expert instructors and coaches.

Others have suggested that the use of models of effective practice, such as modeling instruction or providing samples of lesson plans, example student work, or video case examples, are an important characteristic of effective PD [42]. Perceived relevance—including teachers’ individual perceptions of how PD aligns with their own and their schools’ goals, as well as teacher involvement in the planning of the PD—may also matter for its effectiveness [46,48]. In practice, however, despite these research-based recommendations for effective PD in academic instruction, teachers’ PD experiences regarding teaching academic content are generally described as inadequate, fragmented, and not aligned with these suggested best practices [42,45,47]. Most teachers continue to receive PD in a workshop format of short duration, with an average of about eight hours spent on a topic [42]. 

Although research has been conducted regarding teacher PD for academic instruction, relatively little research has examined the characteristics of effective PD activities and processes for teachers as they relate to effective SMHP implementation [13,14,15]. Identifying what works in teacher PD for SMHP, while maintaining an awareness of the practicalities of the school context, may increase the use of effective PD practices in this area which, in turn, may increase the likelihood that teachers will effectively implement and sustain the implementation of SMHP innovations [19]. Additionally, given the constraints present in schools (e.g., the school calendar, teacher schedules, other teacher responsibilities, budgeting), it is also important that PD be efficient. PD needs to be good enough to help teachers reach a level of implementation integrity that leads to results within the limits of the available resources [15]. This research is also relevant to broader questions about how best to assess and ensure quality of training and TA in schools and in other settings [49]. Thus, research to clarify the specific components that are effective as well as necessary for supporting teachers’ PD for SMHP implementation is critical in moving toward more effective and efficient PD practices. 

### 1.5. Purpose of Current Review

The purpose of this paper is to conduct a systematized literature review to examine the components of effective teacher PD for implementation of SMHP innovations. While SMHP touches every aspect of classroom life, in particular this review focuses on PD for specific universal prevention innovations (i.e., programs, practices) being introduced into a school setting. This review will give guidance for providers of teacher PD for SMHP, for schools embarking on implementation of SMHP innovations, and researchers studying SMHP and SMHP implementation. Specifically, the review will address the following questions:

1. What are the components of high-quality teacher PD for SMHP that lead to effective implementation of innovations?
2. What is the evidence base for these components?

Based on this review, we put forth a summary of recommended practices for teacher PD for SMHP to support teachers in enacting SMHP innovations effectively; recommendations regarding strategies to increase the presence of these best PD practices in schools; and suggested future research needed in this area.

## 2. Materials and Methods

The online databases PsychINFO and ERIC were searched for relevant texts. The search primarily focused on articles from refereed professional journals due to the quality assurance built into the peer review process. Government reports and relevant books were also reviewed. The search was conducted using the combinations of the following keywords: “professional development” OR “technical assistance” OR “coaching” OR “teacher training”, to capture a broad range of PD activities; the keywords “school mental health” OR “social and emotional learning” OR “character education” OR “universal prevention”, to capture some of the range of SMHP innovations (see [8] regarding the breadth of terms encompassed within the concept of school mental health); AND “school OR teacher.” Using these search criteria, 387 total records were identified. Of these, 37 were exact duplicates and were removed, leaving 350 remaining records for further screening. Records needed to meet the following eligibility criteria for further review: (1) substantially focused on PD for a SMHP prevention program or practice within a school setting in an English-speaking country; (2) focused on the PD of teachers, rather than on the PD of school mental health professionals or other school staff or TA provided at the organizational (school) level; and (3) not solely focused on school athletic programs or religious education. Based on these criteria, 83 full-text documents were obtained and reviewed. Of these, texts were retained if they (1) provided a description of a theory, model, or framework regarding effective teacher PD for SMHP or (2) contained a direct empirical examination of one or more components of PD for SMHP, using qualitative or quantitative methodology. Nineteen texts providing a description of a theory, model, or framework and 15 texts presenting a direct, empirical examination of one or more components of PD for SMHP were ultimately retained and included in the following review. These texts were analyzed in two phases, beginning with the descriptive texts and then comparing these results to the empirical texts. 

Phase 1. Potentially effective components of teacher PD for SMHP were first identified through a review of the set of descriptive papers. This review was conducted using the Dedoose Version 8.2 online application for qualitative and mixed methods research. An inductive approach was used to generate the initial codes, which were iteratively reviewed and revised to generate a set of effective components of PD suggested within these texts. Dedoose provides support for an iterative process through which the initial codes were developed and tagged to text excerpts; as more literature was reviewed and excerpts were tagged, these codes were combined and refined. After generating an initial list of codes for potential components, the first author reviewed the content and number of excerpts associated with each code to identify the level of support and areas of potential overlap. Components that were referenced in fewer texts and substantially overlapped with other identified components were combined into a single code to simplify and enhance the usability of the resulting synthesis. For example, codes such as “teacher attitudes”, “buy-in”, and “resistance” tagged on a first reading of several papers were then combined into the broader code of “motivation”.

Phase 2. After identifying the components of effective PD within the descriptive literature in Phase 1, the first author reviewed empirical papers examining teacher PD for SMHP, including study design and outcomes. This analysis employed a deductive approach, applying the codes generated in Phase 1, while also allowing for the emergence of additional codes. The goal of this analysis was to directly link relevant empirical evidence to components of effective PD for SMHP identified during Phase 1. 

## 3. Results

**Figure 1:**
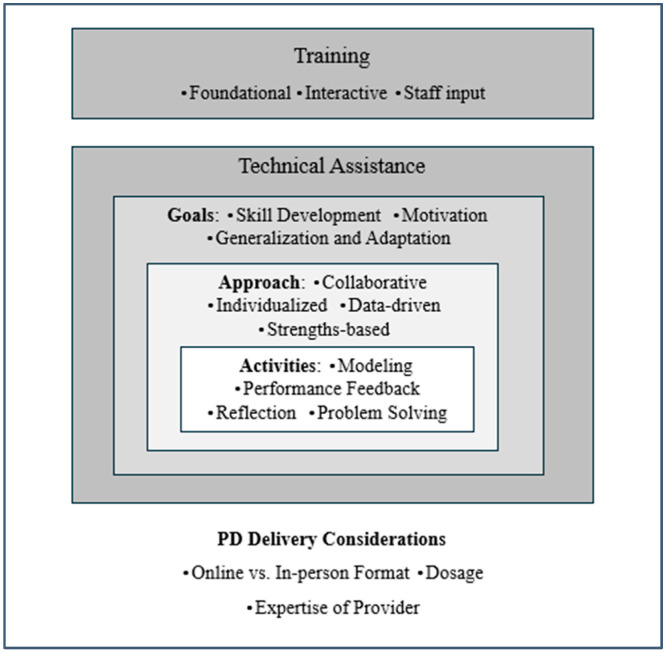
Summary of components of professional development for school mental health promotion.

In alignment with past literature, the models put forth for effective PD for SMHP included both training and TA. While some specifics were included regarding training, much of the literature reviewed described components of TA, including the goals, approach, and activities involved. The following sections summarize the proposed components of effective training and TA, as well as the empirical evidence relevant to these components. A summary of the components is depicted in Figure 1, with definitions of each component provided in Table 1. Table 2 under Section 3.3.3 displays a summary of the components addressed within each descriptive paper; Table 3 under Section 3.3.3 displays a summary of the components addressed within each empirical paper.

While the initial intention was that the strength of support for the recommended components found in the descriptive literature would be determined by directly linking each component to relevant empirical evidence, the empirical literature was not well aligned with the descriptive models. The empirical evidence largely did not examine specific components of PD, or examined aspects of delivery (i.e., dosage, online platform) that were not directly addressed in the descriptive models. Table 3 displays components addressed in the empirical literature only under Additional Considerations. When specific components were tested, they were often tested as a package, limiting the ability to interpret evidence for any one component. Evidence regarding specific PD components was more often present from survey and qualitative studies of teacher perceptions and preferences, but this evidence was generally not linked to outcomes. While teacher preferences may relate to effectiveness insomuch as training that is extremely disagreeable to the participants is likely ineffective, preference for or satisfaction with a PD component is not a valid measure of effectiveness [50]. Thus, the empirical literature aligned with each component is described, but given the misalignment and limitations in interpretability of this support, overall there is not clear support for the effectiveness of the proposed components of effective PD. Considerations addressed in the empirical literature that were not emphasized in the descriptive literature will be discussed.

### 3.1. Training

Over half of the descriptive papers reviewed referenced training, which refers to session-based professional development, typically at the outset of the adoption of an innovation, to increase teachers’ capacity for implementation. The purpose of this training is to lay the foundation of knowledge, skills, and motivation for teachers to be prepared for program implementation, including the rationale for and components and mechanisms underlying the program or practice [4,15,51,52,53]. This initial training is often described as occurring within a one-time workshop format [13,54]. Importantly, this initial foundational training is noted to be a starting point for implementation and not itself sufficient for the development of the necessary teacher capacities for implementation [4,15,51,55]. 

In nearly every empirical study reviewed, initial training for teachers was present as part of the study procedures. However, almost none of the studies directly tested the impact of this component of PD. Among those that did, Hough [56] found evidence providing some limited support for training, finding that the number of teachers trained in a school was highly correlated with the level of implementation in that school and with the school’s adequate yearly progress, a measure by which schools are held accountable for academic progress under the No Child Left Behind legislation passed in 2001 (No Child Left Behind [NCLB], 2002). In one study testing the immediate impacts of training, Kutcher et al. [57] found that participation in a single-day training session regarding student mental health was associated with improved mental health intervention knowledge and with decreased stigma toward mental illness among teacher attendees, immediately following the training. These studies provide directional support for the benefits of training, but with very limited evidence present, it is not clear from the literature how much participation in initial, foundational training may impact program implementation. 

Among the descriptive papers reviewed, the specifics of foundational training were limited, but two components of effective foundational training were often mentioned: that the training be interactive and that it incorporate staff input. Within the empirical literature reviewed, a similar pattern emerged, with few papers specifically describing the components of training and even fewer directly testing these components. 

#### 3.1.1. Interactive 

While initial training often includes didactic components, some propose that effective training should also include interactive learning. Interactive training requires teachers to actively participate in some way during training rather than passively receiving information. Of the papers reviewed, interactive training included active, hands-on approaches to skill development such as role playing, the observation and analysis of models [15,53,58,59], and opportunities for discussion and reflection [59,60,61]. 

Notably, while an interactive approach to training was sometimes present in descriptions of study procedures, none directly tested the impact of interactive components in training on teacher implementation. An interactive approach component was indirectly tested in a study by Becker et al. [62] which tested the online versus in-person delivery of training. In this study, the in-person training incorporated interactive components, including group discussion and lesson planning, whereas the online training consisted of passively watching a review of the program and a demonstration of quality implementation. Teachers who engaged in the in-person, interactive training did not demonstrate improved attitudes toward the intervention, implementation quality, or intervention delivery frequency relative to those receiving online training. This finding does not support the importance of interactive learning, although there is insufficient evidence to draw conclusions specific to this aspect of training. Studies of teacher preferences provide some support for interactive training approaches. In qualitative research, teachers report wanting training to be very applied to practice [56,63]. In one study in which researchers surveyed 1010 teachers, the majority (77%) tended to prefer a hands-on approach to training workshops [64]. However, a sizable minority (23%) preferred to observe rather than try out new strategies. Thus, there is some indication that many but not all teachers may prefer interactive training. 

#### 3.1.2. Staff Input

Incorporating staff input when designing training may help training be more effective. Staff input regarding the topics to be covered and the alignment of a program or practice with the school’s mission and values can be elicited through a staff survey or whole-school discussion incorporated into a faculty meeting or PD session [51,53,65]. Incorporating the teacher perspective in this way when designing foundational training helps to ensure that the training is valued by teachers and promotes commitment, energy, teacher leadership, and, ultimately, behavior change in adopting new classroom practices [51,52,58,66]. 

While the descriptive papers encouraged staff input, there was very little support for staff input in training within the empirical literature reviewed. Only one paper, in which researchers worked to contextualize an SMHP innovation, even mentioned including staff input in training as part of implementation [63]. In this study, teachers qualitatively perceived the resulting training to be interesting and to enhance their understanding. However, none of the papers reviewed provided direct evidence regarding the impact of this component on teachers’ implementation.

### 3.2. Technical Assistance

All the descriptive papers reviewed included some form of TA as part of effective PD. While TA encompasses a range of ongoing support activities, here TA is used to refer to support targeting the capacity of individual practitioners to deliver an SMHP innovation [13]. In contrast to the components of training, which were only briefly mentioned and received limited description, many papers included more detail regarding components of effective TA. The large majority (15 of 19) of papers specifically described a model of ongoing TA referred to variously as coaching or consultation [4,13,14,15,51,52,53,54,55,59,67,68,69,70,71]. There is not consistency in how these terms are defined in the literature [70]. Both coaching and consultation generally refer to individualized, ongoing PD or guidance to improve the implementation of a program or practice [14,15,55,68]. These two terms are typically used interchangeably, and while some authors suggest differences between the two, there is no consensus on the distinction between the terms or on the activities involved in each [15,55,68,70,71]. For the purposes of this paper, the term coaching will be used for this type of ongoing, individually focused support. In addition, the TA described will primarily be coaching, unless otherwise specified in the text. 

TA was similarly a common component included in the empirical literature, present in all but one of the papers reviewed. Of the papers including TA, all but one [72] included some form of coaching. Several papers directly tested the impact of coaching on SMHP implementation, with mixed results. Sutherland et al. [73] found that teachers who received coaching demonstrated stronger innovation adherence and skill competence. Coaching was associated with improvements in teacher-reported outcomes (i.e., teacher efficacy, student achievement) but not objective outcomes (i.e., academic performance on standardized assessments, attendance, out of school suspension) in a study by Bradshaw et al. [74]. In a study examining coaching plus access to online resources compared to access to online resources only, there were no significant benefits of coaching for teacher-reported student outcomes (i.e., social competencies [75]). Notably, this coaching was provided remotely via online communication. Overall, there are not enough studies directly testing SMHP-related coaching on implementation to draw strong conclusions. Those reviewed suggest a possible impact of coaching on more proximal outcomes such as teacher perceptions and program adherence but provide weaker support for more distal outcomes such as student achievement or student social skills. 

Studies directly assessing teacher preference indicate support for TA and coaching. Cunningham et al. [64] found that the stated preference of a majority of teachers (77%) was to have coaching provided to all participants following initial SMHP training and additional follow-up PD sessions after the initial training. In a mixed methods study, teachers selected coaches as the biggest source of support in survey responses and provided positive qualitative feedback about the value of their coaching experiences in supporting implementation [76]. In a qualitative study of the components included in teachers’ coaching experience, teachers found coaching acceptable and were again generally positive about their experiences [77]. 

Overall, TA, and coaching in particular, were discussed more commonly and in more detail than training. Thus, a number of specific components of TA emerged through this literature review. These components fell within three main areas: goals, approaches, and activities. These components were primarily relevant to TA in the form of coaching. Although these components might also be applicable to PD more generally (i.e., session-based training), they were overwhelmingly described in relation specifically to coaching. Thus, they are included here as components of TA, but researchers and practitioners may also want to consider these components within SMHP PD more generally. 

#### 3.2.1. Goals 

While the overall purpose of TA as part of teacher PD for SMHP is generally to improve implementation, the more specific goals of TA may include developing teacher skills, building motivation and buy-in, and assisting with generalization and adaptation of practices of program components. 

**Skill Development**. The goal of skill development within coaching focuses on building on and enhancing skills initially taught in foundational training to refine these skills and enhance fluency. The goal of skill development was commonly mentioned in both descriptive and empirical papers. However, it was not often directly tested in empirical research. Owens et al. [78] included skill development as a key goal within a multi-component package targeting teacher knowledge, skills, and motivation-relevant attitudes, and found support for this multi-component package only in a subset of teachers with lower knowledge, skills, and motivation at baseline. Given that multiple components within this package were tested simultaneously, it is not possible to isolate the impact of this particular goal. In a survey of teacher preferences, teachers reported that they preferred PD (session-based and coaching) to emphasize skills and teach skills that apply to all students [64]. Thus, while the empirical literature mirrored the descriptive literature in that skill development was a common goal, it is not clear that including skill development as a key goal of TA is associated with stronger implementation. 

**Building Motivation**. Another of the more common goals included in the descriptive papers reviewed was building the motivation of teachers. This goal acknowledges that teachers are not universally eager to adopt new programs and practices, and may experience varying levels of resistance [53,71]. To address teacher motivation, the descriptive literature reviewed suggested that coaches might specifically target teachers’ beliefs and attitudes toward the intervention (e.g., acceptability, alignment with values, appropriateness, expectations of success), self-efficacy, commitment, and resistance [14,53,68,69,71]. Some researchers suggest that incorporating approaches based on models of behavior change, social influence, market research, and motivational interviewing (MI) may be helpful in addressing this goal [14,15,68]. While most papers did not contain more detailed recommendations about how to address this goal in practice, Lee et al. [14] described a detailed coaching model based on this goal, using MI as the framework for coaching strategies (see [79] for MI navigational guide information). 

In the empirical literature reviewed, the goal of enhancing motivation was mentioned and tested several times. Steed and Durand [80] directly compared an experimental coaching approach which addressed teacher attitudes relevant to motivation to “traditional” coaching. The experimental coaching focused specifically on self-efficacy, theorized by the researchers to underlie motivation to adopt new practices. In the motivation-focused model, coaches employed cognitive-behavioral techniques to help teachers explore topics including attitudes, beliefs, self-talk, feelings that arose in the classroom, and assumptions/attributions about students’ behavior. The results supported that this experimental coaching approach positively impacted implementation, with teachers who received motivation-focused coaching implementing more intervention skills and reporting fewer students with significant social and emotional difficulties than teachers receiving “traditional” coaching. In another study comparing coaching which incorporated MI to a “traditional” coaching control, teaching behaviors and classroom atmosphere significantly improved for teachers receiving the MI-based coaching, whereas teachers in the control condition improved only in their teaching behaviors [81]. All teachers receiving the MI-based coaching found this process helpful. It is important to note that, while addressing motivation was the focus of this coaching approach, the overall package included other potentially effective SMHP coaching components (i.e., collaborative, individualized), which cannot be disentangled when interpreting these results. In another study of a multi-component coaching package, which included addressing beliefs related to motivation and buy-in as a key goal of coaching, the multi-component package was associated with an improvement when compared to coaching “as usual” only among a subset of teachers with lower knowledge, skills, and motivation at baseline [78]. 

In these studies, TA providers attempted to influence motivation by changing teachers’ intrinsic motivation (i.e., attitudes and beliefs). In contrast, in a qualitative study of teachers’ coaching experiences, teachers described coaching as an extrinsic motivator [77]. Rather than changing their attitudes or beliefs, coaching provided an external reminder and pressure to enact innovation activities. Similarly, in qualitative data collected in a study of teachers’ use of online TA resources, teachers described an external motivator, compliance with the requirements of study participation, as the primary motivator for them to engage with TA supports [72]. Taken together, the available evidence provides some limited support for addressing motivation and buy-in as a promising TA goal that may improve implementation, and it is possible that the act of a coach showing up or another form of accountability associated with TA may in itself create external motivation for implementation. However, with limited available evidence, it is difficult to draw a strong conclusion regarding the impact of this goal on teachers’ SMHP implementation.

**Generalization and Adaptation**. Generalization and adaptation of the program or practice was also identified as a key goal of coaching in some of the descriptive studies. Generalization and adaptation can include adapting the intervention to fit local needs and challenges, integrating the intervention into the curriculum and routines, helping teachers implement the intervention in alignment with their teaching style, and assisting with the pacing of intervention delivery. In the empirical literature, however, generalization was rarely mentioned as a goal. There were no empirical studies which directly tested the impact of this TA goal on implementation. In one qualitative study of teachers’ coaching experiences, only 2 of 33 teachers described that coaches provided support for generalizing the intervention to wider practice, but these teachers did find this support to be valuable [77]. Overall, the evidence regarding including generalization and adaptation as a goal of TA is extremely limited, and teachers may not perceive this as an important part of their TA experience. With almost no empirical studies regarding this goal, it is not possible to draw conclusions about the impact of including this TA goal on teachers’ SMHP implementation. 

#### 3.2.2. Approach 

In the descriptive literature reviewed, authors suggested that to address the aforementioned goals, the way in which providers approach the process of TA delivery and their relationship with teachers is critical. While specific activities were also described and are summarized in the following section, it is the providers’ approach which underlies the effective delivery of these TA activities. Approach to delivery was discussed within the literature specifically in the TA format of coaching, and thus TA providers will be referred to as coaches. The papers reviewed suggest that coaches should take an approach to TA that is collaborative, individualized, data-driven, and strengths-based. Although these approaches are discussed in isolation, in practice there was commonly overlap in the use of these approaches (e.g., feedback was provided in a manner that was both collaborative and data-driven). 

**Collaborative**. The descriptive papers reviewed strongly encouraged a collaborative approach to SMHP coaching. Lee et al. [14] described that a collaborative approach is the “spirit” of the work of coaching (p. 227), and this idea that coaching is voluntary and collaborative is central to many definitions of coaching [68,69,71]. When taking a collaborative approach, a coach works to establish a safe, supportive, non-hierarchical relationship with the teacher in which they can work together to improve SMHP implementation.

The descriptive literature emphasizes that a collaborative approach may be particularly important early in the SMHP coaching process. Building rapport and a strong working relationship is a first, foundational step of SMHP coaching [14,55]. This can be especially critical as some teachers may feel resistance about coaching [53] and as coaches are likely to be brought in when there is an identified crisis or acute need that may be accompanied by distress [69]. Several concrete early actions coaches can take include the following: providing teachers with assistance in preparing their classroom for the intervention; spending time in and learning about the teachers’ classroom; investing in getting to know the teachers on a personal and professional level by spending time with them (e.g., eating in the teachers’ lounge) and making connections based on shared interests and experiences; and being flexible and cooperative with teachers when scheduling meeting times [53,55]. Another important early action is to clarify the coach’s role and responsibilities [53,55,69]. During this process, it is recommended that the coach acknowledge that the teacher might feel uncomfortable and directly emphasize the collaborative nature of the relationship, providing reassurance that the coach’s role is to provide support and not to evaluate performance [55,69]. Through this role clarification and the coach’s approach, he or she can establish an equal power status early in the relationship [14,68]. An early investment in developing a collaborative relationship may help make coaching more effective.

The literature describes that this collaborative approach carries on throughout coaching. As they help teachers improve SMHP implementation, coaches can avoid criticism by using questioning and thoughtful comments, encouraging self-reflection rather relying on direct feedback, affirming and celebrating what teachers are doing well, and using collaborative language (e.g., “what if we tried…”) as they make recommendations [53,58,70]. By recognizing and respecting teachers’ autonomy, experiences, needs, and priorities as they adopt a new program or practice, coaches can support teachers as teachers themselves choose to make changes [14,53,58]. Teachers also can be directly involved in developing an action plan which guides the coaching process [54]. By using a collaborative approach, coaches can guide and support rather than push teachers to make meaningful changes to support SMHP implementation. 

Although a collaborative approach was emphasized in the descriptive literature, it was not as commonly addressed in empirical research. In one study, a collaborative approach was tested as part of a package of coaching strategies, and teachers receiving this package of coaching strategies demonstrated a significantly improved classroom atmosphere and teaching behaviors, whereas teachers receiving the traditional coaching control improved only in teaching behaviors [81]. In qualitative feedback, teachers who received the more collaborative coaching reported that the encouraging and supportive coaching approach was helpful. However, it is not possible to assess the impact of the collaborative approach on outcomes separately from the other components included in the coaching package. While several other papers explicitly mentioned coaches taking a collaborative approach in their methods section [73,78,80], a collaborative approach to coaching was not directly tested in any of these studies. In one of these studies, teacher feedback included that they found coaching to be supportive and helpful [80]. With such limited research, it is not possible to draw conclusions about the impact of a collaborative approach on SMHP implementation.

Qualitative evidence regarding teachers’ coaching experiences and preferences indicated that teachers found a collaborative approach to be helpful. In one mixed methods study, teachers described that support for implementation came in particular from coaches creating a psychologically safe context, which included providing validation, not rushing their pace of implementation, and providing social support [76]. In another study, teachers described that coaches routinely engaged in providing support, guidance, and validation [77]. The study authors noted validation in particular to be one of the components teachers most valued in their coaching experiences. In another qualitative study, teachers emphasized relationship building and a collaborative approach as important in coaching and PD delivery [63]. Although not always naming a collaborative approach directly, these results support that teachers appreciate coaches approaching their interactions with teachers in a way that is supportive, is validating, and is non-hierarchical. 

**Individualized**. Many of the models reviewed emphasized that SMHP coaching should be individualized to be effective. An individualized approach to coaching focuses on addressing each individual teachers’ needs by providing the appropriate level of support [15,54,55,58,69]. This can include responding to individual teachers’ readiness and motivation for change, as well as other barriers to implementation [14,55]. 

In some cases, individualized may mean individually delivered, while in other cases teachers’ individual needs may be met within a group format [54,69]. Both the level of teacher need, as well as teacher and provider preferences, might be considered in making this determination [54,68,69]; however, there is very limited research or guidance on how to determine the appropriate format and intensity of coaching a teacher may need to support SMHP implementation [55]. Becker et al. [55] provide a model that explicitly lays out a process to differentiate coaching based on individual teacher needs. In this two-phase model, all teachers participate in an initial universal phase focused on the teacher–coach relationship, refining skills, and building motivation for implementation. In the second phase, teachers exhibiting a solid implementation based on data collection are routed to a “consolidate course” of coaching aimed at maintaining implementation quality and skills, while teachers who are resistant to implementation or struggling to implement with quality are routed to an “intensive course” of coaching that includes more frequent coaching aimed at continuing support of implementation and skill development [55] (p. 218). As this model reflects, taking an individualized approach to coaching recognizes that the intensity and activities of coaching are not one-size-fits-all and that to achieve adequate implementation, it is likely most efficient to target more resources toward teachers with the highest level of need. 

A review of the included empirical literature indicates that SMHP coaches often take an individualized approach, and that there may be value in targeting coaching towards teachers based on individual need. The description of an individualized approach was explicitly present in several studies [59,62,63,73]. Several other studies examined tailoring coaching based on teacher need. In one study, teachers were selected for the study based on low implementation quality, to mirror the teachers most likely in a real-world setting to be selected for coaching [81], and coaching was associated with improved performance for all of these teachers. In another study, which allowed coaching frequency to vary for a portion of the coaching relationship, an analysis of coaching frequency suggested that coaches were providing a differential frequency of coaching, and that this frequency was associated with specific teacher characteristics [70]. Specifically, teachers receiving a high frequency of coach contacts reported more negative beliefs and perceptions (i.e., burnout, efficacy) at the start of the year compared to low-contact teachers. Teachers receiving a low contact frequency implemented intervention activities with less frequency than moderate- or high-contact teachers, but reported more positive perceptions of organizational health and less burnout. Due to the correlational nature of these data, directional relationships between these characteristics and coaching implementation cannot be established, but these data do suggest that coaches tailor their coaching behaviors based on individual teacher characteristics. In another study, an experimental coaching package was found to improve teachers’ SMHP skills relative to “traditional” coaching only for the subset of teachers beginning with lower knowledge, intervention-supportive beliefs, and implementation skills at baseline [78]. This finding suggests that tailoring coaching, such that coaches provide more intensive coaching to teachers with a lower readiness based on an assessment of individual need, may be most efficient for improving implementation. Taken together, the individualization of coaching is likely happening and may be an avenue for improving coaching efficiency, but additional research is needed to clarify the relationship between individualization and teacher SMHP implementation quality. 

**Data-driven**. The descriptive literature emphasized a data-driven approach to SMHP coaching, in which coaches collect and use data throughout the coaching process to guide their work. Data may be collected through formal and informal interviews and classroom observations [14,55]. Coaches may also use tools such as implementation guidelines and rubrics to help structure data collection [55]. 

Data collection may vary over the course of coaching. Early in coaching, data collection tends to focus on identifying the needs, strengths, and values of the teacher and classroom [14,55,68]. These data may inform the focus of coaching, with coaches and teachers using data to identify, prioritize, and address areas for improvement [14,54,55,58]. For example, coaches and teachers may collaboratively set data-based goals and develop an action plan aligned with these goals to help guide and focus coaching activities [54,55]. As coaching progresses, data may help coaches and teachers assess performance and progress. Coaches may use data sharing as an integral part of performance feedback to teachers [14,55,60]. Ongoing data collection may also help coaches to provide specific guidance regarding classroom practices [54]. For example, Hemmeter et al. [54] describe a model structured around the use of the Teaching Pyramid Observation Tool (TPOT), an observation measure designed to capture the implementation of a set of universal SMHP classroom practices. Using observable indicators, coaches are able to capture implementation quality and provide support to increase fidelity. Thus, a data-driven approach may help coaches and teachers throughout the coaching process to develop a shared focus for coaching and target work toward implementation improvement. 

Using data can also help with the efficient use of coaching capacity [54,55,60]. Data collection focused on implementation quality and intervention effectiveness can help coaches to identify teachers who may need more intensive support [14,54,55,68]. Data may also help coaches to select appropriate strategies and support for individual teacher needs [54]. Thus, data collection allows for more precision in both the intensity/frequency and activities of coaching, increasing efficiency. 

The methods in many of the empirical papers reviewed described a data-based approach [62,70,72,73,74,78,81], but there was no study which tested a data-based approach directly. In the large majority of empirical studies that included a data-based approach, coaches used formal rubrics to assess implementation as part of the coaching process [62,70,73,78,81]. In one study, implementation reports based on teacher and student data were available for teachers to access through an online platform [72]. However, these reports were not reviewed with a coach, and teacher use depended on each teacher’s initiative. In qualitative follow-up interviews with participating teachers, the large majority (13/15) of interviewees reported that they only briefly looked at these reports but did not use them to enhance implementation. The two teachers who did use these reports described an important role for these data in self-monitoring and enhancing implementation fidelity. In one qualitative study, coaching providers (i.e., community mental health professionals) who were included with teachers in qualitative data collection reported that “concrete and clear” observation forms, as well as other materials and procedures to structure coaching, would be helpful in keeping coaching focused and productive ([63], p. 493). Overall, the empirical literature supported that a data-driven approach is likely common in coaching, may be useful for teachers who are motivated to engage with these data to enhance fidelity, and may be helpful in keeping coaching focused. However, no research reviewed directly tested the impact of data collection and use in coaching on teachers’ SMHP implementation. 

**Strengths-based**. Several models included an explicit focus on SMHP coaches and TA providers taking a strengths-based approach to enhance the collaborative teacher–coach relationship [14,55,58]. A strengths-based approach is reflected through behaviors including keeping a positive focus, explicitly acknowledging and affirming teacher efforts and strengths, and the recognition and celebration of success. 

A strengths-based approach may be especially helpful when providing feedback. For example, when discussing performance, coaches can explicitly provide feedback on strengths and incorporate acknowledgement of persistence, effort, and competence in reflective statements during feedback conversations [14,55]. Becker et al. [55] recommend beginning feedback conversations with teacher strengths before providing constructive feedback on growth areas. Coaches can also focus on specific observable behaviors to avoid making negative attributions about shortcomings in implementation [55]. Verbal praise, written notes posted in the classroom, individual rewards, and public recognition of teachers and teams within the school are also strengths-based approaches that can enhance teachers’ experience of support from coaches and strengthen the relationship [55]. Taking steps to acknowledge and celebrate teacher strengths and progress can help build a solid foundation for collaboration between coaches and teachers. 

In the empirical literature reviewed, a strengths-based approach was present in the methods for one experimental study [78] and in two qualitative studies [63,77]. In the qualitative research, teachers commented that coaches’ feedback promoted confidence and that they particularly appreciated that coaches presented even corrective feedback in a positive and supportive way [77]. The qualitative feedback also highlighted that discussing effective moments before challenging ones and positive behaviors before difficulties was a helpful way for coaches to address teacher skepticism and defensiveness [63]. 

Overall, the empirical literature is very limited in this area, but the qualitative studies provide some indication that teachers may appreciate a collaborative approach that feels supportive and validating. In particular, teachers may feel more comfortable and supported when coaches take a strengths-based approach to feedback, and this could provide a context for collaboration. However, there is no evidence available to clarify the impact of this approach on teachers’ implementation of SMHP innovations. 

#### 3.2.3. Activities

The activities of TA are specific actions and behaviors TA providers, including coaches, employ to move teachers toward TA goals, conducted in alignment with the approach to TA described above. There is not clarity or consensus in the literature on the activities coaches should employ to improve teacher implementation of SMHP interventions or how these activities should be structured and delivered [55,60]. However, among the descriptive papers reviewed, several key activities were most often identified: modeling, performance feedback, reflection, and problem solving. These activities were described, and thus will be discussed primarily, within the TA format of coaching. The descriptive papers reviewed offer some specifics regarding these coaching activities, but how to best sequence and engage in these activities and the relative effectiveness of these strategies is not well studied [15,55,59]. The activities selected may vary depending on the context, needs, and preferences of those involved [15,69]. In addition, the selection and focus of these activities typically varies across the course of coaching as goals and needs shift [14,15,53,55]. Thus, while the activities described below are commonly agreed upon as important for effective coaching, the specifics of how and when these activities should occur is not clear and likely varies based on individual needs and across the course of coaching. 

In the empirical literature, these activities were also often present, and they arose in qualitative descriptions of and preferences for coaching. However, the majority of these activities were not directly tested with regard to their impact on teacher implementation. Thus, it is not possible to draw strong conclusions from this literature about the impact of these activities on teacher implementation nor on the specifics of when and how these activities might be most effectively implemented during coaching. 

**Modeling**. Among the descriptive papers reviewed, several resources specifically discussed modeling the delivery of SMHP program skills and activities as a key coaching activity. This allows teachers to observe the effective implementation and proper use of program materials [14,55]. Hamre et al. [75] extended the concept of modeling beyond direct modeling by coaches, suggesting that teachers observe and analyze other models (e.g., colleagues, video models) as part of ongoing TA. While most papers provided few specifics regarding the best approach to modeling, Becker et al. [55] described that coaches may want to provide teachers with the opportunity to try out the modeled skill as soon as possible after the skill is modeled by the coach, at which time the coach should be available to provide support and assist the teacher as needed. 

In the empirical literature reviewed, modeling was mentioned in the methods section of several papers. Several of these referred vaguely to modeling without any specifics [62,70,72]. One study specified that coaches could use the modeling of an agreed-upon skill to begin their in-class coaching session [73]. In several other papers, modeling referred primarily to video modeling [72,75,78,81]. In some cases, support was provided for teachers to learn from these video models. In one study, processing this video model was completed together with a coach [78]. In another, text appeared on screen to highlight important aspects of implementation occurring within the video clip [75]. In one qualitative study, teachers described that modeling was present in their coaching experiences [77]. While part of the coaches’ explicit role within the innovation was to model the innovation, the paper described that this did not occur for most teachers, although they thought it would have been helpful. Among those teachers who did experience modeling, they felt it was helpful because it was good to see the intervention modeled, to observe students’ responses, and to validate that they were implementing the innovation correctly. 

Modeling was directly tested in one empirical study, which examined engagement with video modeling in relation to student outcomes and also compared the outcomes associated with video modeling versus video modeling plus video coaching [75]. In this study, teachers who spent more time using web-based video modeling reported greater increases in student social competence. However, due to the study design, it is not possible to determine whether this was a result of teachers engaging with the video models or due to a third variable (e.g., higher motivation) that increased both the use of the video models and the implementation quality. Additionally, in this study, there was no benefit to student social competencies with the addition of coaching, suggesting that in this case, coaching may not have significantly improved implementation above the effects of video modeling. In another study, guided video modeling that included an analysis of strengths and weaknesses was a key activity used to enhance teachers’ skill development [78]; however, because it was tested as part of a package of coaching practices, the impact of this particular component cannot be determined. These findings do not provide clear evidence for or against the benefits to implementation quality of modeling as part of SMHP TA. 

Studies of teacher preferences provide some support for modeling and specifically reveal a preference for modeling specific to the individual’s classroom context. In one qualitative study, teachers perceived modeling to be one of the components of coaching most important to enhancing implementation [76]. Teachers specifically described that watching the coaches model implementation within the teachers’ own classrooms helped them to both believe that the innovation could work in their classrooms and to better understand what implementation should look like in that setting. In another qualitative study, teachers reported that it was helpful for coaches to demonstrate the new practices that they introduced [63]. They also shared that they wanted more video modeling as part of PD and for these video models to include more ethnic minority students to reflect their classroom demographics. These results suggest that teachers may find modeling helpful and in particular prefer modeling that helps them envision implementation within their own classroom setting. However, none of the empirical results address the impact of modeling on teacher implementation behaviors. 

**Observation and performance feedback**. The descriptive resources included a cycle of observation and performance feedback as a central element of effective SMHP coaching. This includes coaches repeatedly observing a teacher’s skills in delivering an SMHP innovation and providing constructive feedback focused on improving implementation. In addition to assisting in the development of innovation-specific skills, the models reviewed assert that this cycle of feedback may help with the generalization and integration of skills into teaching practices, the promotion of teacher self-monitoring, and a reduction of decline in implementation quality over time [55,68,71]. 

The descriptive papers that discussed feedback suggested that this feedback be targeted. For example, Hemmeter et al. [54] described the approach of “practice-based coaching”, which includes the explicit identification of practices, performance goals, and a plan of action to improve upon those practices to provide a focus for coaching (p. 206). Becker et al. [55] suggested prioritizing and focusing feedback and future coaching on the one or two areas of improvement that are likely to be most impactful in the classroom, rather than developing a more exhaustive list of areas of implementation to address. 

Tools such as performance measures and rubrics may be helpful for coaches in guiding the process of observation and feedback delivery, making them both more targeted and more effective [4,14]. The use of these tools allows for consistency in coaching guidance. Coaches may share these tools, as well as data collected with these tools, with the teacher to help facilitate collaborative discussion about performance. Sharing measurement tools directly with teachers creates a common language and goals that coaches can use to acknowledge improvements in performance. Sharing data derived from these tools can also facilitate feedback delivery. Lee et al. [14] suggest that it is useful for coaches to enter into feedback conversations with teachers by presenting the data that were collected (e.g., providing a graphic display of performance data), and asking teachers to first provide their impressions and interpretation of the data collected before the delivery of feedback by the coach, creating a more collaborative feedback conversation.

The delivery of performance feedback is also an opportunity to develop teachers’ motivation for change and to highlight and build on strengths [14,55]. Lee et al. [14], in their paper describing a motivational interview-based approach to coaching, suggest a set of steps to guide sharing performance feedback that include the following: (1) gathering information on the teacher’s impressions of the data, (2) providing affirmative statements regarding the teacher’s autonomy in and commitment to change, (3) observations and reflection that highlight strengths and competencies, and (4) exploration with the teacher of gaps between the teacher’s values and goals and implementation-related actions. Thus, in addition to building skills and awareness, this cycle of observation and feedback may also present opportunities to build motivation to make and maintain implementation improvements. 

Observation and feedback in various forms were commonly present in the empirical literature reviewed, but this component was not directly tested. While studies did not provide insight into the effectiveness of observation and feedback as part of SMHP coaching, they did provide some description of the format of this component. In some studies, coaches conducted live, in-person observations with real-time or immediate feedback [62,70,73,78,80]. In one study, teachers sent in video tape of implementation for coaches to “observe”, and coaches provided detailed written feedback and occasional video chat [75]. In another study, implementation reports were available for teachers to access online but were not reviewed with a coach [72]; these reports were accessed very infrequently, although the teachers who did access them found them helpful in improving implementation. Thus, observation and feedback are common within TA for SMHP and may take a variety of forms, but it is not known how this component of TA may impact teachers’ implementation. 

The qualitative studies provided some limited support for observation and feedback having a positive impact on implementation. In one study, teachers described that coaches conducted observations based on a fidelity checklist [77]. They reported feeling apprehensive at first about the process of observation and feedback but ultimately found it to be a positive and helpful component of coaching. These teachers perceived that the coaches were providing feedback to help rather than to judge them and described that the coaches often provided validation. Ultimately, they felt that this observation and feedback could help improve their teaching practices. One teacher in this study described diminishing returns of the observation/feedback cycle over time, suggesting that this component of coaching was most helpful early on in skill acquisition. In another study, teachers also indicated that it was helpful for coaches to observe and provide feedback particularly as they were learning new practices [63]. These qualitative results indicate that teachers may perceive some benefit of the process of observation and feedback on implementation and, after initial discomfort, find it to be acceptable and helpful. 

**Reflection**. Within the descriptive literature reviewed, reflection was described as an important element of TA. Reflection includes teachers taking time to consider their experiences and behaviors within their classroom, as well as the impact of classroom practices. Engaging in reflection helps teachers to use their practical experiences, to enhance awareness and knowledge of their teaching practices and to self-initiate change [58,59,61]. Thus, reflection itself can be viewed as a form of PD that enhances teacher classroom practices and teacher effectiveness [58,61]. This process of teacher self-reflection can be enhanced by a coach’s outside perspective [58,59]. 

To encourage reflection, coaches should develop a reflective and supportive relationship with teachers and embed reflection within ongoing coaching activities [52,54,70]. The focus of reflection can shift over the course of coaching. Early in the coaching relationship, teacher reflection on current practices can help to identify areas for improvement [14,54]. If using a practice-based coaching model, as described previously, this early reflection can help guide the development of a plan of action to focus coaching activities on priority areas [54]. As coaching progresses, coaches can encourage reflection on the teacher’s implementation performance as well as on student response to the teacher’s implementation [55]. By incorporating teacher reflection on performance, coaches can encourage teacher insight and performance improvement while preserving a collaborative relationship. Reflection connecting implementation and student outcomes can help teachers to make a connection between the innovation and student behavioral progress, which can help to build motivation and teacher self-efficacy [55]. Reflective conversations also allow coaches to assess teachers’ enthusiasm or identify and address ambivalence [55]. Thus, reflection can serve a variety of useful purposes across the course of coaching, including supporting initial goal setting, improving implementation performance, and building teacher motivation. 

In the empirical literature, reflection was mentioned in the methods of several papers [63,73,80], but was not directly tested nor present in qualitative studies of SMHP TA. Reflection was one component of a package of coaching components tested in one study [78], and in this study, teachers with lower knowledge, skills, and supportive beliefs at baseline showed improvement when then received coaching with these additional components compared to teachers who received coaching as usual. It is not possible to draw conclusions about the impact of the use of reflection in SMHP coaching from this study. Thus, while reflection was commonly included in models of SMHP coaching, with very little research and no direct test of this component, it is not possible to draw conclusions about its impact on teacher implementation. 

**Problem solving**. Another coaching activity included within the descriptive literature reviewed was problem solving, which includes working to identify and address challenges and barriers to implementation [15,55]. Problem solving within coaching can take a variety of forms. Problem solving in some cases is described as making up a large focus of TA [15,55,68,69] while in others it is included on an as-needed basis [52,55,60]. Coaches may integrate problem solving into coaching through a systematic and ongoing problem-solving process, including data collection, problem identification and modeling, planning, and ongoing evaluation [55,68,69]. Problem identification can occur throughout coaching, within cycles of observation and feedback [55]. Problem solving can also be brief, occurring as part of regular check-ins and focusing on a minor barrier or specific challenge [55,60]. While it may vary in intensity and form, problem solving may help improve implementation by addressing challenges that arise as teachers take on a new program or practice. 

Problem solving was also mentioned in several empirical papers but was never directly tested, nor did it explicitly present in qualitative studies of teachers’ experiences and preferences. While problem solving was commonly included in the descriptive literature, there was no evidence for this component within the empirical literature reviewed. Thus, it is not known how this component of SMHP coaching impacts teacher implementation based on the reviewed research. 

### 3.3. PD Delivery Considerations

In the review of empirical literature, several additional logistical considerations not emphasized in the descriptive literature emerged regarding SMHP PD, relevant to both initial training and TA. These considerations did not emerge as key components of effective teacher PD for SMHP in the descriptive literature reviewed but were directly studied in the empirical literature. These considerations include online vs. in-person format, the expertise of the coach, and dosage. 

#### 3.3.1. Online vs. In-Person Format 

Teacher PD for SMHP can occur via workshops or coaching from an in-person provider or can be conducted online. Online PD can occur either using self-directed resources with built-in feedback mechanisms or through online correspondence with a PD provider. In the descriptive literature, this format consideration was directly mentioned in only a few papers, although it appeared that most papers assumed an in-person model. Owens et al. [15] mentioned the possible advantages of an online format, including efficiency; individualization; and flexibility with regard to timing, scheduling, and delivery. This paper also pointed out the research opportunity this type of online individualization might present for clarifying what works when and for whom in SMHP teacher PD. In contrast, Becker et al. [55] pointed out several possible disadvantages of online TA as compared to in-person coaching: the availability of video observation only, no classroom modeling and prompting, and more limited possibilities for individualization. Finally, Chafouleas et al. [51] suggested that the use of online training resources is helpful only as an initial step, to be supplemented by future coaching. Thus, support was mixed for an online SMHP PD format the few times it was mentioned within the descriptive literature.

Although largely absent in the descriptive literature, an online format for teacher PD for SMHP was directly tested in several empirical papers, also with mixed support. In one study, online training was compared to in-person training; in both conditions, teachers received in-person coaching [62]. In this study, there were no significant differences in teacher attitudes toward the intervention, implementation quality, or frequency of intervention delivery between teachers receiving online as compared to in-person training, and a survey of teacher perceptions indicated no difference in the perceived effectiveness of the two formats. This suggests that an online format for initial training may be as effective as in-person training, if in-person coaching is also provided. 

Other studies examined an online format for TA delivery. In one study, researchers found that the provision of online resources (video models) resulted in the same increases in students’ social competencies as the provision of these resources paired with online coaching [75]. They further found that the increased use of these online resources by teachers was associated with greater gains in their students’ social competence. These results indicate that access to online resources may be helpful and may even be as helpful as online coaching in some cases, but it is not possible to draw conclusions from these data about the relative effectiveness of in-person versus online TA. Another study examined self-directed online training and TA (i.e., online support resources, including implementation quality reports [72]). The dosage of online training and the initiation of online support requests both significantly predicted teachers’ adherence, controlling for their baseline capacity. Their adherence, in turn, significantly predicted their students’ social and emotional outcomes. However, in qualitative data collection, teachers indicated that they took little initiative and had low motivation to access online resources. They preferred non-technology options for resource access when possible and described their primary motivation for accessing these online resources as study compliance. Very few teachers regularly accessed implementation quality reports to improve practices. Thus, teachers did not demonstrate a high level of initiative to independently access online resources and support. While the use of these online resources was associated with some positive outcomes, it is likely the teachers accessing these resources more frequently differed in other meaningful ways from teachers with a lower frequency of access. In another study surveying teacher preferences regarding PD, teachers did not prefer an online format [64]. Teachers in this study who indicated more readiness to adopt new SBMP innovations did indicate some willingness to engage in internet-based learning activities, but about a quarter of the sample, who expressed lower readiness, indicated that they would prefer not to engage in any internet-based PD activities.

Taken together, these results provide some preliminary support for initial training in an online format but present a more mixed picture of an online format for ongoing support. While providing access to online resources may be helpful, it is not clear that teachers will independently take advantage of ongoing support available in an online format, nor how ongoing support available online compare to in-person coaching. However, given continuing advances in the relevant technology and the advantages an online format may have for efficiency, flexibility, and individualization, it is likely research in this area will continue.

#### 3.3.2. Dosage

Optimal dosage—the amount of time and exposure to training and TA needed to achieve the desired level of implementation—of teacher SMHP PD was not directly addressed in the descriptive literature but was a topic of study in a number of the empirical papers. Two descriptive papers did include detailed dosage suggestions. The dosage of coaching was central in the model proposed by Becker et al. [55]. In this model, initially all teachers receive regularly scheduled weekly or biweekly coaching sessions for approximately 4–6 weeks to build rapport and enhance skills development. This is followed by 10–15 min weekly or biweekly check-ins for the remainder of the year for most teachers, with longer and more intensive, individualized coaching sessions for teachers struggling with implementation. Eppler-Wolff et al. [53] also include weekly coaching sessions in their model, recommending 20–30 min but recognizing that practical constraints may dictate 10–15 min sessions. Also included in their model, coaches spend half-days in classrooms at the outset of the coaching relationship, and teachers participate in “regular” PD workshops along with weekly coaching [53] (p. 12). Other papers were more vague in their suggested dosage, stating that coaching should be of an adequate time and should occur in an ongoing manner until the outcomes are achieved [60,69]. Two papers also mentioned that the existing research has not systematically evaluated the duration and intensity needed to achieve adequate implementation levels [15,60]. Thus, the descriptive literature provides little concrete guidance around dosage decisions. 

Information on dosage was provided in the large majority of empirical papers, and was an area of study in several. For training, dosage ranged from one 8 h day [57,62,73] to two full weeks [76], most commonly falling somewhere between one and three days. In some studies, initial training was followed by a full- or half-day booster session [70,81]. Coaching was typically conducted in 20–40 min sessions occurring weekly to biweekly for between 6 and 14 weeks [62,63,73,78,80], and up to a full school year [74,81]. 

Several empirical studies examined dosage. In a pre–post study, Kutcher et al. [57] found that a one-day, 8 h training session was sufficient to increase teacher knowledge and reduce stigma toward mental health as measured following the training. A study of teacher preferences found that teachers prefer this type of 1-day workshop training format [64]. However, another study measuring implementation-related outcomes found that a longer duration was needed to see results [56]. In this study, teachers participated in PD including one week of training and intermittent coaching. This PD was associated with increased confidence and implementation quality after 2 years but not after 1 year of involvement, indicating that longer-term PD may be most effective. These results indicate that teachers may prefer 1-day workshops and that this training format may lead to some immediate learning. However, a change in classroom practices and the more distal impacts of these changes may require longer-term involvement in PD. 

Several studies examined the relationship between TA dosage and implementation, with mixed results. Pas et al. [70] found that coaching dosage was unrelated to implementation quality but did relate to teacher attitudes and implementation frequency. Teachers initially demonstrating high burnout and lower efficacy received more coaching, while those receiving less frequent coaching reported a more positive perception of organizational health and lower burnout at the end of the year. Two other studies measured teachers’ use of online TA materials and found a significant relationship between this usage and outcomes. Hamre et al. [75] found that teachers who spent more time using online support materials (i.e., web-based video modeling) reported greater increases in their students’ social competence. Livet et al. [72] found that the dosage of online training completed and the use of student progress reports available as an online support resource were significant predictors of teacher adherence to the innovation, over and above baseline teacher capacity. Teacher adherence, in turn, predicted student social and emotional outcomes, although these outcomes were not directly predicted by the dosage of online TA access. However, results from a survey of teacher preferences support the possibility that pre-existing teacher characteristics may determine the observed dosage, when dosage is determined by teacher initiation of TA [64]. In this study, three-quarters of more change-ready teachers indicated that they would like SBMP PD to include 2 1 h follow up workshop-style PD sessions and ongoing coaching, while a subset of teachers who reported less readiness for change preferred to have no follow-up TA unless it was teacher-initiated.

Taken together, these studies suggest that dosage of TA could relate to implementation quality. However, because dosage was allowed to vary freely and was observed rather than randomly assigned, it is not possible to draw conclusions about the directionality of the results or to make firm recommendations about how to determine the optimal dosage. 

#### 3.3.3. Expertise of the Provider

Another consideration that arose in empirical and, in particular, in qualitative research regarding teacher PD for SBMP was the expertise of the PD provider. In the empirical literature reviewed, expertise was generally defined by teachers’ perception of the provider’s expertise, although it is likely that expertise relates to who the providers are (e.g., experience, education, internal/external staff) and their preparation for the provider role (e.g., experience, training, and ongoing supervision). All of these characteristics likely relate to the perceived and actual skills of the PD provider, both of which may impact effectiveness. 

In the descriptive literature, the quality of facilitation was occasionally mentioned [15,58]. Several articles provided some suggestions regarding who might provide PD, including skilled internal staff or community members with experience with the innovation or who might be trained as part of a train-the-trainer approach (e.g., school psychologists, social workers, counselors, administrators) or external providers (e.g., graduate students in psychology; [4,14,15,53]). Owens et al. [15] indicated that provider selection is an area in need of research. Two papers provided more detailed information regarding provider preparation. Lee et al. [14] in their model emphasized the importance of training and ongoing PD for internal staff to act as coaches, beyond what is typical for staff within an educational setting, recommending two full-day workshops and ongoing supervision. They specifically described the need for this PD to be tailored based on these providers’ prior experiences, including building general capacities and the innovation-specific capacities required within their model for teacher training and TA. Similarly, Eppler-Wolff et al. [53] described a model using psychology graduate students as TA providers, in which the students received two 8-h training workshops, including a focus on general and innovation-specific capacities, as well as ongoing supervision by licensed psychologists for 2 h per week. 

The empirical literature often described the qualifications of the PD providers. Most commonly, these were external providers, often university-affiliated research staff, which included graduate and postdoctoral students or former teachers. Several studies included a mention of the training and supervision of the PD providers, with supervision generally occurring biweekly or monthly. Training was rarely specified, although in one paper in which PD was provided by community mental health providers, the paper specified that providers attended 3 half-day training sessions [63]. In surveys of teacher preferences and qualitative studies, teachers indicated a preference for skilled, expert trainers [64,76]. In one qualitative study of teachers’ coaching experiences, the study authors suggested teachers’ responses revealed a perception that their coaches were skilled providers and indicated that this was important in their experience of coaching [77]. Overall, it appears that researchers are attentive to the qualifications of providers in the context of research studies and that the perceived expertise of the provider matters to teachers. However, it does not appear that there is clarity in the literature about what provider characteristics or preparation is necessary for them to deliver PD for SMHP that positively impacts teachers’ implementation. 

**Table 2:** Descriptive articles reviewed and components included.

				Goals				Approach				Activities			
Study	Training	Staff Input	Interactive	Technical Assistance	Develop Skills	Motivation	Generalization/Adaptation	Collaborative	Individualized	Data-driven	Strengths-Based	Modeling	Observation/Feedback	Reflection	Problem Solving
Becker et al., 2013 [55]	X			X	X	X		X	X	X		X	X	X	X
Berkowitz, 2011 [65]	X	X	X	X											
Chafouleas et al., 2016 [51]	X	X		X	X										
Domitrovich et al., 2012 [67]				X									X	X	
Edgar, 2013 [58]		X	X	X				X	X					X	
Elias, 2008 [52]	X	X		X		X								X	X
Eppler-Wolff et al., 2019 [53]	X	X	X	X		X	X	X							
Erchul, 2015 [68]				X	X	X	X	X	X	X		X	X		X
Flaspohler et al., 2006 [13]	X			X											
Gibson et al., 2014 [69]	X			X	X	X		X	X						X
Hamre et al., 2012 [75]	X		X	X								X		X	
Hemmeter et al., 2018 [54]	X			X				X	X	X			X	X	
Lee et al., 2014 [14]				X		X		X	X	X		X	X	X	
Owens et al., 2014 [15]	X	X	X	X	X	X			X				X		X
Pas et al., 2015 [70]		X		X	X		X	X		X		X	X	X	X
Schultz et al., 2015 [71]				X	X	X		X					X		X
Silva & Gimbert, 2001 [61]				X										X	X
Swain-Bradway et al., 2015 [4]	X			X	X		X			X			X		
Vetter, 2008 [66]		X		X											

Note. X indicates component was present in paper, as determined by explicit mention or description of component within model.

**Table 3:** Empirical articles reviewed and components included.

				Goals				Approach				Activities				Other Considerations		
Study	Foundational Training	Staff Input	Interactive	Technical Assistance	Develop Skills	Motivation	Generalization/Adaptation	Collaborative	Individualized	Data-driven	Strengths-Based	Modeling	Observation/Feedback	Reflection	Problem Solving	Online	Dosage	Expertise of Provider
Ashworth et al., 2018 [77]				Q		Q	Q	Q			Q	Q	Q				X	Q
Becker et al., 2014 [62]	T		T	X	X				X			X	X		X	T	X	
Bradshaw et al., 2012 [74]	T			T	X					X					X		X	
Cappella et al., 2011 [63]	Q	Q	Q	X				Q	X	Q	Q	Q	Q	X			X	
Cunningham et al., 2013 [64]	P		P	P	P												P	
Hamre et al., 2012 [75]	X			T					X			T	X		X	P	T	
Hough, 2011 [56]	T		P	X								P					T	P
Kutcher et al., 2013 [57]	T		X														X	
Livet et al., 2018 [72]	X			X						X		X	X			Q	T	
Owens et al., 2017 [78]	X			P	T	T		X	T	X	X	T	X	T	X		X	
Pas et al., 2015 [70]	X			T					T	X		X	X				T	
Reinke et al., 2012 [81]	X		X	X	X	T		T	T	X	X	X	X		X		X	
Steed & Durand, 2013 [80]				X	X	T		X					X	X	X		X	
Sutherland et al., 2015 [73]	X			T	X			X	X	X		X	X	X	X		X	
Wanless et al., 2013 [76]	X			Q				Q				Q	X			X	Q	

Note. X indicates component was present in paper, as determined by explicit mention or description of component within model; T indicates component was tested in study; Q indicates component was addressed in qualitative data in study; P indicates teacher preference regarding component that was assessed in study.

## 4. Discussion

Building teachers’ capacity for implementing SMHP innovations through high-quality PD is critical to successful implementation. In this review of the literature, recommendations regarding SMHP PD within the descriptive and empirical literature converged on some common PD components. While these components provide an important starting point for better understanding how to evaluate the quality of training and TA provided to schools [49], further empirical evidence is needed to support the impact of these components on teachers’ SMHP implementation. 

The amount and quality of evidence available to address this review’s research questions is one major limitation of this review. Few components have been directly tested; rather, many were tested as part of a package, were observed rather than experimentally tested, or were studied based on teacher preference. Notably, this is in part related to the interrelated nature of these components within the practice of TA provision. For example, a strengths-based approach may contribute to more successful collaboration; observation and performance feedback can be enhanced by a data-driven approach; a strengths-based approach may be central to work on the goal of enhancing motivation. Thus, while these components are distinct, in practice they may be difficult to isolate and study. In addition, while there may be agreement about helpful PD components, in both the descriptive and empirical literature there is often a lack of specifics regarding how the component should be implemented. Thus, the existing literature cannot support conclusions about the contributions of specific PD practices in achieving successful implementation and outcomes. 

Other limitations to these results relate to decisions about the scope of the literature included in this systematized review. This review included articles yielded by a limited set of search terms, but given the breadth of terminology used to describe SMHP activities [8], it is possible this methodology resulted in missing some relevant papers. For example, though this paper focused on preventative interventions, broadening the search terms to include other classroom practices (e.g., “classroom management”) that often can include preventative strategies may have yielded additional relevant results. The set of literature reviewed might also be expanded by combing the introduction of each paper for the literature cited and evaluating relevant citations for inclusion. Based on descriptions of past research cited within the papers reviewed, this approach would likely yield some additional studies and in particular might strengthen the empirical literature base included. The current review also specifically synthesizes studies of PD for teachers within the realm of SMHP. However, because activities of PD occur across professional fields and may be informed by other fields of study (e.g., behavior change, adult learning), this approach may miss potentially beneficial PD components that have not traditionally been included in models and studies of teacher PD for SMHP. Finally, because the scope of literature selected in this review focuses on research specifically describing PD rather than on SMHP program implementation more broadly, it is not possible to draw conclusions regarding the extent to which these components are applied in SMHP implementation outside of research specifically describing or examining PD.

Despite these limitations, the components identified represent promising practices in teacher PD for SMHP, based on the available literature. All the components that emerged from the descriptive literature were studied in at least one empirical paper, with many consistently present across empirical studies. Many of the components were associated with some positive outcomes and had a perceived value to teachers. Thus, while the literature reviewed does not allow for strong conclusions regarding the effectiveness of these components on SMHP program outcomes, the components that emerged appear to be representative of a current standard of practice in SMHP PD for teachers.

### 4.1. Future Directions: Research to Practice

It is clear that additional research is needed to determine the PD components most effective in improving teachers’ SMHP implementation. Research is also needed to better clarify, if possible, when and for whom these components may be effective. This could take the form of experimental research employing a stronger study design. For example, designing studies in which PD components are provided to different groups of teachers could help with a more direct comparison to better specify how each component contributes. Moving away from research relying on teachers’ preferences and perceptions as the primary outcome and instead examining changes in behavior (e.g., classroom practices) or more distal outcomes (e.g., student social and emotional competence) could also strengthen our understanding of the impact of these components. Finally, additional research examining specific practices could be helpful for enhancing understanding of how best to enact PD components. Operationalizing more general components (e.g., a collaborative, data-driven approach) into specific practices (e.g., sharing and discussing a visual representation of data collected according to a specific rubric) could allow for more nuanced recommendations about these practices. 

It is also possible that specification of which components matter and under what conditions could be undertaken through a meta-analysis of SMHP innovation research, comparing the effect sizes associated with SMHP innovations in the presence and absence of PD components. However, this would rely on descriptions of PD within the methods presented in these studies. Currently, the methods sections may or may not include clear descriptions of PD. Thus, this line of research might require researchers to use more consistent descriptions of components when describing experimental methods. As this area of research matures, it will be also be helpful to consider how teacher characteristics (e.g., well-being, years of experience, baseline motivation) may moderate the importance of these PD components. 

Based on the literature reviewed, research regarding effective training for teachers in the area of SMHP is particularly needed. Although many descriptive and empirical studies included foundational training as a component, little detail about what this involves was provided. Foundational training may provide teachers a first exposure to an SMHP innovation, and given that it is likely some will be resistant [33,34,64], this training may be an important point for addressing this possible resistance and gaining buy-in. 

Relatedly, it seems that continuing to study how best to achieve the goal of building motivation is an area open for innovation. In the literature on organizational capacity, motivation has been identified as a key component of readiness [82]. It seems that one key difference between PD for SMHP and for academic instruction is that some teachers may not feel that SMHP is within their role and may demonstrate a lack of readiness to adopt SMHP innovations [33,34,64]. In contrast, it seems less likely that a teacher would view academic instruction as outside their scope. Thus, with many of the standards of practice derived from the literature on teacher PD for academic instruction, it seems likely that this component may require unique attention in the context of SMHP PD. Future research should continue to explore and apply what is known about behavior change within the field of psychology, as well as other disciplines, to address motivation and thereby improve teacher classroom practices. 

### 4.2. Future Directions: Practice to Research

The components identified in this review, as a synthesis of the standards of practice in teacher PD for SBMP, have a practical use for those planning PD in this area. While there is a gap between the practices included in models of teacher PD for SMHP and the empirical research supporting those practices, there is likely an even larger gap between these general practice standards and PD taking place within schools. We know that many SMHP innovations in schools fail and are not sustained, and it is likely that inadequate capacity building contributes to this [19,36]. Thus, one possible and important application of these components could be as a framework for planning and evaluating teacher PD for SMHP to improve PD quality. Toward that aim, these components could be transformed into a tool for use by schools and PD providers in planning SMHP innovation implementation. This tool could operate as a checklist or as a menu of options to consider and incorporate into PD planning. This tool could also provide a measure of quality based on alignment with the existing practice standards to promote assessment and improvement of implementation quality.

A tool based on these components could serve the immediate practical purpose of improving the alignment of PD with the practice standards as well as generat important research regarding teacher PD for SMHP. We do not have a good idea of what routinely happens in the provision of PD in schools in the area of SMHP. Regular use of this type of tool could generate a clearer understanding of the state of PD in SMHP. Over time, this type of data collection could allow for research regarding the effectiveness of PD components, as well as clarification about the conditions under which they are effective. Assessing effectiveness using this type of practice-to-research approach could be particularly helpful because, as in research on evidence-based innovations, the key elements of training and TA may be hard to specify and components may operate in tandem, making them hard to disentangle and manipulate individually as in traditional experimental research [36]. Many of the empirical studies reviewed tested training and TA components as a package, which likely reflects the extent to which these components are difficult to disentangle [36]. Thus, this approach might better allow for studying the components of PD for SMHP in a more generalizable format and across various contexts. 

Further, practice-based research should also focus on better understanding promising practices and barriers not well reflected in the existing literature. For example, mixed methods research using a tool designed around these components could allow researchers to learn from PD providers about specific practices associated with these components, as well as about innovative practices not captured in the existing literature. This type of research may also yield valuable information regarding barriers to quality PD and where drift from faithful implementation begins to occur in the process of training and TA. 

Finally, to improve PD quality, future research should include an examination of how adherence to standard practices for SMHP PD for teachers may vary across PD providers. As suggested within the empirical literature, having expert providers of PD is likely an important element in high-quality teacher PD for SMHP [14,15,56,64,76]. Often in research studies, PD is provided by the research team (e.g., [62,72,75]), but this model may not be generalizable. While outside the context of research, schools may sometimes be able to manage PD for SMHP using internal staff as PD providers, such as through train-the-trainer models [15], schools often rely on outside providers to coordinate and implement PD. These providers include federally funded TA centers (e.g., Technology Transfer Centers; Substance Abuse and Mental Health Services Administration, 2019), state-funded regional PD providers (e.g., Educational Service Centers; OESCA, 2019), and nonprofits. Understanding the extent to which PD conducted by different internal and external providers varies and how these variations align with the practice standards might provide important decision-making information to school leaders about PD resource allocation. Information regarding these variations may also be useful for PD providers in improving their practices.

## 5. Conclusions

SMHP has the potential to improve access to preventative intervention and thereby support student mental health, and teachers are often on the front lines of delivering SMHP innovations. Despite limitations within the existing literature on how best to prepare teachers for this role, the components synthesized in this review provide a starting point for designing teacher PD for SMHP based on the available literature. Further, these components may help in designing research to better understand what works in teacher PD for SMHP and to develop tools to learn from what is happening in the field. As our knowledge of effective PD practices continues to improve, we may be able to develop approaches and develop policy to improve PD for teachers in the area of SMHP and, ultimately, to improve mental health outcomes in schools and communities.

## Figures and Tables

**Table 1:** Description of components of effective professional development for school mental health promotion.

Component	Description
Training	Workshop to provide introduction to innovation and build needed knowledge, skills, and motivation
Staff input	Gather input (e.g., whole staff survey) regarding topics to be covered and alignment of innovation with school mission
Interactive	Employ active approaches to skill development (e.g., role playing, observation, analysis of models) and include opportunity for discussion and reflection (e.g., connect to current practices)
Technical Assistance	Ongoing professional development or guidance to improve the implementation of a program or practice, often coaching
Goals	The overall purpose of TA as part of teacher professional development for SMH is generally to improve implementation
Developing teacher skills	Enhance and refine skills acquired in training
Building motivation	Address teacher beliefs and attitudes toward the intervention, self-efficacy, commitment, and resistance to increase likelihood of behavior change
Assisting with generalization and adaptation	Help teachers adapt innovation to fit local needs and challenges and to integrate with existing curriculum, routines, schedule, and teaching style
Approach	The TA providers’ approach underlies effective delivery of TA activities
Collaborative	Work with teachers as partners by building rapport and shared understanding of goals, challenges, and progress
Individualized	Tailor frequency, intensity, and focus of support to address unique needs and challenges of different teachers
Data-drive	Use formal and informal data to identify needs, strengths, and values to best prioritize goals and assess implementation quality
Strengths-based	Acknowledge and celebrate strengths, effort, and success; build on strengths before providing feedback on growth areas
Activities	The activities of TA, undertaken using the above approaches, help to achieve TA goals
Modeling	Directly model innovation-specific skills, or provide and analyze video models of these skills
Observation and performance feedback	Repeatedly observe implementation in person or using video recording and provide detailed, targeted feedback
Reflection	Incorporate reflection throughout TA, using collaborative reflection to identify areas of focus for coaching and encourage teacher insight into progress and areas for improvement
Problem solving	Identify and address barriers through a systematic problem-solving process

## Data Availability

The articles reviewed are outlined in Table 2 and Table 3 and within the References. Further inquiries can be directed to the corresponding author.
